# Effectiveness of Maternal mRNA COVID-19 Vaccination During Pregnancy Against COVID-19–Associated Hospitalizations in Infants Aged <6 Months During SARS-CoV-2 Omicron Predominance — 20 States, March 9, 2022–May 31, 2023

**DOI:** 10.15585/mmwr.mm7239a3

**Published:** 2023-09-29

**Authors:** Regina M. Simeone, Laura D. Zambrano, Natasha B. Halasa, Katherine E. Fleming-Dutra, Margaret M. Newhams, Michael J. Wu, Amber O. Orzel-Lockwood, Satoshi Kamidani, Pia S. Pannaraj, Katherine Irby, Aline B. Maddux, Charlotte V. Hobbs, Melissa A. Cameron, Julie A. Boom, Leila C. Sahni, Michele Kong, Ryan A. Nofziger, Jennifer E. Schuster, Hillary Crandall, Janet R. Hume, Mary A. Staat, Elizabeth H. Mack, Tamara T. Bradford, Sabrina M. Heidemann, Emily R. Levy, Shira J. Gertz, Samina S. Bhumbra, Tracie C. Walker, Katherine E. Bline, Kelly N. Michelson, Matt S. Zinter, Heidi R. Flori, Angela P. Campbell, Adrienne G. Randolph, Overcoming COVID-19 Investigators, Candice Colston, Heather Kelley, Meghan Murdock, Laura Miron, Ronald C. Sanders, Daniel Hakimi, Jaycee Jumarang, Kennis-Grace Mrotek, Liria Muriscot Niell, Natasha Baig, Lexi Petruccelli, Yamila Sierra, Elizabeth Temte, Imogene Thayer, Frances Zorensky, Nadine Baida, Jong-Ha C. Choi, Caroline R. Ciric, Mark D. Gonzalez, Bria M. Coates, Heather E. Price, Mary Stumpf, Maya Clark, Rylie Dittrich, Suden Kucukak, Eve Listerud, Patrick Moran, Noelle M. Drapeau, Brandi A. Johnson, Lacy Malloch, Lora Martin, Maygan Martin, Kayla Patterson, Cameron Sanders, Shannon Pruitt, Children’s Mercy Kansas City, Melissa Sullivan, Stephanie P. Schwartz, Merry Tomcany, Chelsea C. Rohlfs, Amber Wolfe, Fatima A. Mohammed, J. Nelson Reed, Zachary Rusler, Jack Thomas, Cayla Wakser, Kailee Fernandez, Laura S. Stewart, Leenah Abojaib, Molly J. Kyles

**Affiliations:** ^1^Coronavirus and Other Respiratory Viruses Division, National Center for Immunization and Respiratory Diseases, CDC; ^2^Department of Pediatrics, Vanderbilt University Medical Center, Nashville, Tennessee; ^3^Department of Anesthesiology, Critical Care and Pain Medicine, Boston Children’s Hospital, Boston, Massachusetts; ^4^The Center for Childhood Infections and Vaccines of Children’s Healthcare of Atlanta and the Department of Pediatrics, Emory University School of Medicine, Atlanta, Georgia; ^5^Division of Infectious Diseases, Children’s Hospital Los Angeles, Los Angeles, California; ^6^Department of Pediatrics, University of California, San Diego, San Diego, California; ^7^Section of Pediatric Critical Care, Department of Pediatrics, Arkansas Children’s Hospital, Little Rock, Arkansas; ^8^Department of Pediatrics, Section of Critical Care Medicine, University of Colorado School of Medicine, Aurora, Colorado; ^9^Children’s Hospital Colorado, Aurora, Colorado; ^10^Department of Pediatrics, Division of Infectious Diseases, University of Mississippi Medical Center, Jackson, Mississippi; ^11^Division of Pediatric Hospital Medicine, University of California San Diego-Rady Children’s Hospital, San Diego, California; ^12^Immunization Project, Department of Pediatrics, Baylor College of Medicine, Texas Children’s Hospital, Houston, Texas; ^13^Division of Pediatric Critical Care Medicine, Department of Pediatrics, University of Alabama at Birmingham, Birmingham, Alabama; ^14^Division of Critical Care Medicine, Department of Pediatrics, Akron Children’s Hospital, Akron, Ohio; ^15^Division of Pediatric Infectious Diseases, Department of Pediatrics, Children’s Mercy Kansas City, Kansas City, Missouri; ^16^Division of Pediatric Critical Care, Department of Pediatrics, University of Utah, Salt Lake City, Utah; ^17^Primary Children’s Hospital, Salt Lake City, Utah; ^18^Division of Pediatric Critical Care, University of Minnesota Masonic Children’s Hospital, Minneapolis, Minnesota; ^19^Department of Pediatrics, University of Cincinnati College of Medicine, Cincinnati Children’s Hospital Medical Center, Cincinnati, Ohio; ^20^Division of Pediatric Critical Care Medicine, Medical University of South Carolina, Charleston, South Carolina; ^21^Department of Pediatrics, Division of Cardiology, Louisiana State University Health Sciences Center, New Orleans, Louisiana; ^22^Children’s Hospital of New Orleans, New Orleans, Louisiana; ^23^Division of Pediatric Critical Care Medicine, Children’s Hospital of Michigan, Central Michigan University, Detroit, Michigan; ^24^Divisions of Pediatric Infectious Diseases and Pediatric Critical Care Medicine, Department of Pediatric and Adolescent Medicine, Mayo Clinic, Rochester, Minnesota; ^25^Division of Pediatric Critical Care, Department of Pediatrics, Cooperman Barnabas Medical Center, Livingston, New Jersey; ^26^Ryan White Center for Pediatric Infectious Disease and Global Health, Department of Pediatrics, Indiana University School of Medicine, Indianapolis, Indiana; ^27^Department of Pediatrics, University of North Carolina at Chapel Hill Children’s Hospital, Chapel Hill, North Carolina; ^28^Division of Pediatric Critical Care Medicine, Nationwide Children’s Hospital Columbus, Ohio; ^29^Department of Pediatrics, Northwestern University Feinberg School of Medicine, Chicago, Illinois; ^30^Division of Critical Care Medicine, Ann & Robert H. Lurie Children’s Hospital of Chicago, Chicago, Illinois; ^31^Department of Pediatrics, Divisions of Critical Care Medicine and Allergy, Immunology, and Bone Marrow Transplant, University of California San Francisco, San Francisco, California; ^32^Division of Pediatric Critical Care Medicine, Department of Pediatrics, C.S. Mott Children’s Hospital, Ann Arbor, Michigan; ^33^Department of Anaesthesia, Harvard Medical School, Boston, Massachusetts; ^34^Department of Pediatrics, Harvard Medical School, Boston, Massachusetts.; Children’s of Alabama, Birmingham, Alabama; Children’s of Alabama, Birmingham, Alabama; Children’s of Alabama, Birmingham, Alabama; Arkansas Children’s Hospital, Little Rock, Arkansas; Arkansas Children’s Hospital, Little Rock, Arkansas; Children’s Hospital Los Angeles, Los Angeles, California; Children’s Hospital Los Angeles, Los Angeles, California; Children’s Hospital Los Angeles, Los Angeles, California; Children’s Hospital Los Angeles, Los Angeles, California; Children’s Hospital Colorado, Aurora, Colorado; Children’s Hospital Colorado, Aurora, Colorado; Children’s Hospital Colorado, Aurora, Colorado; Children’s Hospital Colorado, Aurora, Colorado; Children’s Hospital Colorado, Aurora, Colorado; Children’s Hospital Colorado, Aurora, Colorado; Emory University School of Medicine and Children’s Healthcare of Atlanta, Atlanta, Georgia; Emory University School of Medicine and Children’s Healthcare of Atlanta, Atlanta, Georgia; Emory University School of Medicine and Children’s Healthcare of Atlanta, Atlanta, Georgia; Emory University School of Medicine and Children’s Healthcare of Atlanta, Atlanta, Georgia; Ann & Robert H. Lurie Children’s Hospital of Chicago, Chicago, Illinois; Ann & Robert H. Lurie Children’s Hospital of Chicago, Chicago, Illinois; Riley Hospital for Children, Indianapolis, Indiana; Boston Children’s Hospital, Boston, Massachusetts; Boston Children’s Hospital, Boston, Massachusetts; Boston Children’s Hospital, Boston, Massachusetts; Boston Children’s Hospital, Boston, Massachusetts; University of Michigan; C.S. Mott Children’s Hospital, Ann Arbor, Michigan; Mayo Clinic, Rochester, Minnesota; Mayo Clinic, Rochester, Minnesota; Children’s Hospital of Mississippi, Jackson, Mississippi; Children’s Hospital of Mississippi, Jackson, Mississippi; Children’s Hospital of Mississippi, Jackson, Mississippi; Children’s Hospital of Mississippi, Jackson, Mississippi; Children’s Hospital of Mississippi, Jackson, Mississippi; Kansas City, Missouri; Children’s Mercy Kansas City, Kansas City, Missouri;; University of North Carolina at Chapel Hill, Chapel Hill, North Carolina; Akron Children’s Hospital, Akron, Ohio; Cincinnati Children’s Hospital, Cincinnati, Ohio; Nationwide Children’s Hospital, Columbus, Ohio; Medical University of South Carolina Children’s Health, Charleston, South Carolina; Medical University of South Carolina Children’s Health, Charleston, South Carolina; Medical University of South Carolina Children’s Health, Charleston, South Carolina; Medical University of South Carolina Children’s Health, Charleston, South Carolina; Medical University of South Carolina Children’s Health, Charleston, South Carolina; Monroe Carell Jr. Children’s Hospital at Vanderbilt, Nashville, Tennessee; Monroe Carell Jr. Children’s Hospital at Vanderbilt, Nashville, Tennessee; Texas Children’s Hospital and Baylor College of Medicine, Houston, Texas; Texas Children’s Hospital and Baylor College of Medicine, Houston, Texas

**Summary**

**What is already known about this topic?**

Infants aged <6 months are not eligible for COVID-19 vaccination and are at risk for COVID-19–associated complications. Maternal vaccination received during pregnancy could protect infants from COVID-19–related hospitalization.

**What is added by this report?**

During the period of recent SARS-CoV-2 Omicron predominance, maternal receipt of an mRNA COVID-19 vaccine during pregnancy reduced the likelihood of COVID-19-related hospitalizations and serious complications among infants aged <6 months.

**What are the implications for public health practice?**

Expectant mothers should remain current with COVID-19 vaccination to protect themselves and their infants from hospitalization and severe outcomes associated with COVID-19.

## Abstract

Infants aged <6 months are not eligible for COVID-19 vaccination. Vaccination during pregnancy has been associated with protection against infant COVID-19–related hospitalization. The Overcoming COVID-19 Network conducted a case-control study during March 9, 2022–May 31, 2023, to evaluate the effectiveness of maternal receipt of a COVID-19 vaccine dose (vaccine effectiveness [VE]) during pregnancy against COVID-19–related hospitalization in infants aged <6 months and a subset of infants aged <3 months. VE was calculated as (1 – adjusted odds ratio) x 100% among all infants aged <6 months and <3 months. Case-patients (infants hospitalized for COVID-19 outside of birth hospitalization and who had a positive SARS-CoV-2 test result) and control patients (infants hospitalized for COVID-19–like illness with a negative SARS-CoV-2 test result) were compared. Odds ratios were determined using multivariable logistic regression, comparing the odds of receipt of a maternal COVID-19 vaccine dose (completion of a 2-dose vaccination series or a third or higher dose) during pregnancy with maternal nonvaccination between case- and control patients. VE of maternal vaccination during pregnancy against COVID-19–related hospitalization was 35% (95% CI = 15%–51%) among infants aged <6 months and 54% (95% CI = 32%–68%) among infants aged <3 months. Intensive care unit admissions occurred in 23% of all case-patients, and invasive mechanical ventilation was more common among infants of unvaccinated (9%) compared with vaccinated mothers (1%) (p = 0.02). Maternal vaccination during pregnancy provides some protection against COVID-19–related hospitalizations among infants, particularly those aged <3 months. Expectant mothers should remain current with COVID-19 vaccination to protect themselves and their infants from hospitalization and severe outcomes associated with COVID-19.

## Introduction

COVID-19 during pregnancy is associated with adverse pregnancy and neonatal outcomes (*1*). Transplacental transfer of vaccine-induced SARS-CoV-2–specific antibodies has been demonstrated, and severe clinical infant outcomes related to COVID-19 are preventable through maternal vaccination (*2*,*3*). Effectiveness of maternal vaccination against COVID-19–related hospitalization (vaccine effectiveness [VE]) among infants aged <6 months was previously estimated to be 38% for infants hospitalized during the period of the SARS-CoV-2 Omicron variant predominance (December 2021–March 2022) (*4*). This study provides updated estimates of maternal VE among infants aged <6 months and aged <3 months through more recent periods of Omicron subvariant predominance.

## Methods

The Overcoming COVID-19 Network^§^ used a case-control design to assess VE. Methods have been described previously (*4**,**5*). Infants aged <6 months hospitalized^¶^ with acute COVID-19 as the primary reason for admission who received a positive SARS-CoV-2 nucleic acid amplification test (NAAT) or antigen test result (case-patients) across 26 hospitals during March 9, 2022–May 31, 2023, were included. Control patients were infants also hospitalized for an acute COVID-19–like illness but who received a negative SARS-CoV-2 test result by NAAT testing during their hospitalization or within 7 days before hospital admission. The odds of maternal receipt of ≥1 mRNA COVID-19 vaccine dose during pregnancy (second dose or higher) were compared with having received no vaccine doses among mothers of case- and control patients. Critical illness among case-patients was described by maternal vaccination status. Critical illness was defined as an illness requiring life support (i.e., receipt of invasive or noninvasive mechanical ventilation, vasopressors, or extracorporeal membrane oxygenation), or resulting in death. Infants were excluded from the analysis if they were born to mothers who 1) received their most recent dose before pregnancy, 2) received only 1 mRNA vaccine dose during pregnancy with no vaccination before pregnancy, 3) received their most recent vaccine dose within the 14 days before delivery, 4) received only 1 dose of a viral vector vaccine, or 5) had unknown or unverifiable vaccination timing or status. During the surveillance period, Omicron BA.1/BA.1.1, BA.2, BA.4, BA.5, BQ.1/BQ1.1, XBB.1.5, and XBB.1.16 were the most commonly circulating subvariants.

Maternal vaccination status was ascertained among those who had received ≥2 mRNA vaccine doses, at least one of which occurred during pregnancy, or 1 viral vector vaccine dose followed by ≥1 mRNA vaccine dose during pregnancy. Maternal vaccination status was categorized as 1) unvaccinated (never received COVID-19 vaccine before their infant’s delivery) or 2) vaccinated during pregnancy (receipt of a second or higher dose of either a licensed mRNA vaccine, such as BNT162b2 [Pfizer-BioNTech] or mRNA-1273 [Moderna], or a single dose of Ad.26.CoV2.S [Janssen {Johnson & Johnson}] recombinant vaccine before or during pregnancy and ≥1 mRNA vaccine dose during pregnancy). Timing of vaccination was based on the date of receipt of the most recent vaccine dose. The interval between receipt of the last dose and the infant’s hospitalization was calculated as the number of inclusive days between those events.

VE was calculated as (1 – adjusted odds ratio) x 100% among all infants aged <6 months. Odds ratios were calculated using multivariable logistic regression, comparing the odds of maternal receipt of a COVID-19 vaccine dose during pregnancy with the odds of being unvaccinated between case- and control patients. All models controlled for infant age (in months), sex, race and ethnicity, U.S. Census Bureau region, and month and year of hospital admission.** Generalized estimating equations were used to include study site as a repeated effect. In a secondary analysis, VE among infants aged <3 months was evaluated. Results were not adjusted for multiple comparisons. All analyses were performed using SAS software (version 9.4; SAS Institute). This activity was reviewed by CDC, deemed not research, and was conducted consistent with applicable federal law and CDC policy.^††^

## Results

Among 1,076 eligible infants hospitalized during March 9, 2022–May 31, 2023, a total of 360 (33%) were excluded, 288 (80%) of whom were born to mothers who received their most recent vaccine dose before pregnancy.^§§^ Among the remaining 716 hospitalized infants (377 case-patients and 339 control patients), the median age was 2.3 months (IQR = 1.2–4.2 months), 153 (21%) were reported to have at least one underlying health condition, and 162 (23%) were born before 37 completed gestational weeks (preterm). Among the 377 case-patients, 82 (22%) were born to mothers who had received a COVID-19 vaccine dose during pregnancy, compared with 94 (28%) born to mothers of control patients (p = 0.06) (Table 1). Vaccinated mothers of case- and control patients were similar in terms of timing of vaccine receipt, with approximately two thirds in each group receiving their most recent vaccine dose during the first 20 weeks of pregnancy (p = 0.18). Case- and control patients were similar in age (60% and 63% aged <3 months, respectively; p = 0.42), sex (41% and 45% female, respectively; p = 0.28), race and ethnicity (p = 0.41), U.S. Census Bureau region (p = 0.38), prevalence of preterm birth (24% and 22%, respectively; p = 0.51), and the presence of at least one underlying health condition (23% and 20%, respectively; p = 0.33). The prevalence of underlying cardiac conditions was higher among case-patients (9%) than among control patients (5%) (p = 0.04).

**TABLE 1:** Characteristics of infants* aged <6 months hospitalized with a COVID-19–like illness and a positive SARS-CoV-2 test result (case-patients) or a negative SARS-CoV-2 test result (control patients) — 26 pediatric hospitals, 20 states,^†^ March 9, 2022–May 31, 2023

Characteristic (no. missing)	Case-patients no. (column %) n = 377	Control patients no. (column %) n = 339	p-value^§^
**Median age, mos (IQR)**	2.4 (1.2–4.3)	2.2 (1.2–3.9)	0.17
**Age group, mos**			
0–2	227 (60.2)	214 (63.1)	0.42
3–5	150 (39.8)	125 (36.9)	
**Sex, female **	155 (41.1)	153 (45.1)	0.28
**Race and ethnicity**			
Asian, non-Hispanic	12 (3.2)	10 (2.9)	0.41
Black or African American, non-Hispanic	84 (22.3)	58 (17.1)	
White, non-Hispanic	154 (40.8)	150 (44.2)	
Hispanic or Latino, any race	79 (21.0)	66 (19.5)	
Other, non-Hispanic	24 (6.4)	24 (7.1)	
Unknown	24 (6.4)	31 (9.1)	
**Median SVI (IQR)^¶^**	0.5 (0.4–0.7)	0.5 (0.4–0.7)	0.97
U.S. Census Bureau region**			
Northeast	27 (7.2)	29 (8.6)	0.38
Midwest	80 (21.2)	80 (23.6)	
South	189 (50.1)	148 (43.7)	
West	81 (21.5)	82 (24.2)	
**Omicron subvariant (predominant period of admission)^††^**			
BA.1.1/BA.2 (Mar 9, 2022–Jul 16, 2022)	65 (17.2)	73 (21.5)	0.53
BA.4/BA.5 (Jul 17, 2022–Dec 3, 2022)	146 (38.7)	121 (35.7)	
BQ.1.1 (Dec 4, 2022–Jan 28, 2023)	96 (25.5)	85 (25.1)	
XBB.1.5/XBB.1.16 (Jan 29, 2023–May 31, 2023)	70 (18.6)	60 (17.7)	
**Underlying health condition in infants (1)**			
At least one underlying condition (1)	86 (22.8)	67 (19.8)	0.33
Respiratory condition (1)	27 (7.2)	20 (5.9)	0.50
Cardiac condition (1)	35 (9.3)	18 (5.3)	0.04
Other health condition (1)^§§^	63 (16.7)	48 (14.2)	0.35
**Codetection with respiratory syncytial virus (204)^¶¶^**			
No. positive/Total no. tested	43/262 (16.4)	121/250 (48.4)	<0.01
Preterm birth (<37 wks’ gestation)***	89 (23.6)	73 (21.5)	0.51
**Maternal vaccination^†††^**			
Unvaccinated	295 (78.2)	245 (72.3)	0.06
Vaccinated during pregnancy	82 (21.8)	94 (27.7)	
**Timing of maternal vaccination during pregnancy** ^§§§,¶¶¶^			
Early pregnancy (first 20 wks)	55 (67.1)	62 (66.0)	0.18
Late pregnancy (21 wks–14 days before delivery)	27 (32.9)	32 (34.0)	
**No. of maternal doses received during pregnancy** ^¶¶¶^			
Completed primary series****	22 (26.8)	32 (34.0)	0.30
Received ≥1 booster dose**^††††,^**^§§§§^	60 (73.2)	62 (66.0)	

**Abbreviation:** SVI = social vulnerability index.

* Infants were excluded from analysis if they were born to mothers who had received their most recent dose before pregnancy, received only 1 dose of an mRNA vaccine, received their most recent vaccine dose within 14 days of delivery, received only 1 dose of a viral vector vaccine, or whose vaccination status could not be verified or timing of which was unknown.

^†^ Infants were enrolled from 26 pediatric hospitals in 20 states, in all four U.S. Census Bureau regions. *Northeast*: Boston Children’s Hospital (Massachusetts) and Cooperman Barnabas Medical Center (New Jersey); *Midwest*: Akron Children’s Hospital (Ohio), Children’s Hospital Medical Center (Ohio), Children’s Hospital of Michigan (Michigan), Children’s Mercy Kansas City (Missouri), C.S. Mott Children’s Hospital (Michigan), Lurie Children’s Hospital (Illinois), Mayo Clinic (Minnesota), Minnesota Masonic (Minnesota), Nationwide (Ohio), and Riley Hospital for Children (Indiana); *South*: Arkansas Children’s Hospital (Arkansas), Children’s of Alabama (Alabama), Children’s Healthcare of Atlanta, Emory (Georgia), Children’s Hospital of New Orleans (Louisiana), Medical University of South Carolina Children’s Health (South Carolina), Monroe Carell Jr. Children’s Hospital at Vanderbilt (Tennessee), Texas Children’s Hospital (Texas), University of Mississippi Medical Center (Mississippi), and University of North Carolina at Chapel Hill Children’s Hospital (North Carolina); *West*: Children’s Hospital Colorado (Colorado), Children’s Hospital Los Angeles (California), University of California, San Francisco Benioff Children’s Hospital (California), University of California San Diego-Rady Children’s Hospital (California), and Primary Children’s Hospital (Utah).

^§^ Testing for statistical significance was conducted using the Pearson’s chi-square test and Fisher’s exact test for comparisons with fewer than five observations. Wilcoxon rank-sum tests were used to compare continuous data.

^¶^ Median SVIs for case-patients and control patients are based on 2020 U.S. SVI data. The SVI ranges from 0 to 1.0, with higher scores indicating greater social vulnerability. https://www.atsdr.cdc.gov/placeandhealth/svi/documentation/SVI_documentation_2020.html

** https://www2.census.gov/geo/pdfs/maps-data/maps/reference/us_regdiv.pdf

^††^ Based on CDC’s genomic surveillance system; variant predominance based on first day of the week a subvariant comprised >50% of SARS-CoV-2 specimens. https://covid.cdc.gov/covid-data-tracker/#variant-summary

^§§^ Other health conditions included neurologic or neuromuscular disorders, non-oncologic immunosuppressive disorders, active or previous oncologic disorders, endocrine disorders, diabetes, obesity, rheumatologic or autoimmune disorder, hematologic disorder, renal or urologic dysfunction, gastrointestinal or hepatic disorder, metabolic or confirmed or suspected genetic disorder, or atopic or allergic condition.

^¶¶^ Testing for respiratory syncytial virus was missing or not conducted for 31% of case-patients and 26% of control patients.

*** Missing or unknown prematurity status was classified as term (≥37 weeks’ gestation); six case-patients and eight control patients were missing gestational age and classified as being born at term (≥37 weeks’ gestation).

^†††^ Maternal vaccination status was based on the last date of a COVID-19 mRNA vaccine dose: unvaccinated was defined as mothers who had not received any vaccine dose before or during pregnancy, and vaccinated was defined as mothers who received their last dose of a COVID-19 mRNA vaccine between the first day of pregnancy and 14 days before delivery. Among those vaccinated during pregnancy, mothers could have received ≥1 dose during pregnancy. Mothers could receive 1 dose of Ad.26.CoV2.S (Janssen [Johnson & Johnson]) vaccine before or during pregnancy and 1 dose of an mRNA vaccine during pregnancy. Mothers who received only 1 dose of an mRNA vaccine were considered partially vaccinated and were excluded from the analysis. Mothers whose last vaccine dose occurred before pregnancy were excluded from the analysis.

^§§§^ Timing of vaccination is based on date of receipt of the last dose of a COVID-19 vaccine during pregnancy.

^¶¶¶^ Percentages calculated among those vaccinated during pregnancy.

**** Thirty-six women (17 mothers of case-patients and 19 mothers of control patients) initiated and completed a 2-dose mRNA series during pregnancy.

^††††^ Seven women (three mothers of case-patients and four mothers of control patients) had received a Janssen vaccine dose before pregnancy and an mRNA vaccine during pregnancy; three women (two mothers of case-patients and one mother of a control patient) received 1 Janssen and 1 mRNA vaccine dose before pregnancy, and 1 mRNA vaccine dose during pregnancy; two women (one mother of a case-patient and one mother of a control patient) received all 3 mRNA vaccine doses during pregnancy.

^§§§§^ Eight women received a bivalent dose (five mothers of case-patients and three mothers of control patients).

The median interval between receipt of the most recent vaccine dose and infant hospitalization was 236 days (Table 2). VE of ≥1 COVID-19 vaccine dose during pregnancy against COVID-19–related hospitalizations among infants aged <6 months was 35% (95% CI = 15%–51%). Among infants aged <3 months, VE was 54% (95% CI = 32%–68%), with a median interval between maternal vaccine dose and infant hospitalization of 219 days.

**TABLE 2:** Effectiveness* of a maternal COVID-19 vaccine dose^†^ during pregnancy against COVID-19–associated hospitalization in infants^§^ aged <6 months and <3 months — 26 pediatric hospitals, 20 states,^¶^ March 9, 2022–May 31, 2023

Age group, mos	No. vaccinated/Total no. (%)		Interval between last vaccine dose and infant hospitalization, days (IQR)	VE, % (95% CI)
	Case-patients	Control patients		
0–5	82/377 (21.8)	94/339 (27.7)	236 (185–300)	35 (15–51)
0–2	43/227 (18.9)	63/214 (29.4)	219 (152–264)	54 (32–68)

**Abbreviation:** VE = vaccine effectiveness.

* VE estimates were based on odds of maternal vaccination during pregnancy in case-patients versus control patients, adjusted for U.S. Census Bureau region, admission date (monthly), age (in months), sex, and race and ethnicity (non-Hispanic Black or African American, non-Hispanic White, non-Hispanic other, Hispanic or Latino of any race, or unknown). Study site was included as a repeated effect. VE was calculated as (1 – adjusted odds ratio) x 100%.

^†^ Maternal vaccination status was based on the last date of a COVID-19 mRNA vaccine dose: unvaccinated was defined as mothers who had not received any vaccine dose before or during pregnancy, and vaccinated was defined as mothers who received their last dose of a COVID-19 mRNA vaccine between the first day of pregnancy and 14 days before delivery. Among those vaccinated during pregnancy, mothers could have received ≥1 dose during pregnancy. Mothers could receive 1 dose of Ad.26.CoV2.S (Janssen [Johnson & Johnson]) vaccine before or during pregnancy and 1 dose of an mRNA vaccine during pregnancy. Mothers who received only 1 dose of an mRNA vaccine were considered partially vaccinated and were excluded from the analysis. Mothers whose last vaccine dose occurred before pregnancy were excluded from the analysis.

^§^ Infants were excluded from analysis if they were born to mothers who had received their most recent dose before pregnancy, received only 1 dose of an mRNA vaccine, received their most recent vaccine dose within 14 days of delivery, received only 1 dose of a viral vector vaccine, or whose vaccination status could not be verified or whose timing of vaccination was unknown.

^¶^ Infants were enrolled from 26 pediatric hospitals in 20 states, in all four U.S. Census Bureau regions. *Northeast*: Boston Children’s Hospital (Massachusetts) and Cooperman Barnabas Medical Center (New Jersey); *Midwest*: Akron Children’s Hospital (Ohio), Children’s Hospital Medical Center (Ohio), Children’s Hospital of Michigan (Michigan), Children’s Mercy Kansas City (Missouri), C.S. Mott Children’s Hospital (Michigan), Lurie Children’s Hospital (Illinois), Mayo Clinic (Minnesota), Minnesota Masonic (Minnesota), Nationwide (Ohio), and Riley Hospital for Children (Indiana); *South*: Arkansas Children’s Hospital (Arkansas), Children’s of Alabama (Alabama), Children’s Healthcare of Atlanta, Emory (Georgia), Children’s Hospital of New Orleans (Louisiana), Medical University of South Carolina Children’s Health (South Carolina), Monroe Carell Jr. Children’s Hospital at Vanderbilt (Tennessee), Texas Children’s Hospital (Texas), University of Mississippi Medical Center (Mississippi), and University of North Carolina at Chapel Hill Children’s Hospital (North Carolina); *West*: Children’s Hospital Colorado (Colorado), Children’s Hospital Los Angeles (California), University of California, San Francisco Benioff Children’s Hospital (California), University of California San Diego-Rady Children’s Hospital (California), and Primary Children's Hospital (Utah).

Among the 377 case-patients, 86 (23%) were admitted to an intensive care unit (ICU), and 50 (13%) were critically ill and required life support (Table 3). Mothers of 42 (84%) of the 50 critically ill infants were unvaccinated. Invasive mechanical ventilation was more common among case-patients with unvaccinated mothers (25 of 295, 8%) than among those whose mothers were vaccinated during pregnancy (one, 1%) (p = 0.02). Overall, 77% of case-patients had no reported underlying health conditions. When limited to the 291 case-patients without underlying health conditions, patterns were similar: 22% were admitted to an ICU, 13% were critically ill, and invasive mechanical ventilation was more common among those whose mothers were unvaccinated (18, 8%) compared with those who were vaccinated (0) (p = 0.02).^¶¶^

**TABLE 3:** Clinical outcomes and severity among case-patients* aged <6 months hospitalized with COVID-19, by maternal COVID-19 vaccination status^†^ during pregnancy — 26 pediatric hospitals, 20 states,^§^ March 9, 2022–May 31, 2023

Characteristic (no. missing)	Maternal COVID-19 vaccination status, no. (%)			p-value^¶^
	Total	Unvaccinated	Vaccinated	
**All infants**	**377 (100.0)**	**295 (100.0)**	**82 (100.0)**	**—**
Intensive care unit admission	**86 (22.8)**	65 (22.0)	21 (25.6)	0.55
Critical illness**	**50 (13.3)**	42 (14.2)	8 (9.8)	0.36
Invasive mechanical ventilation	**26 (6.9)**	25 (8.5)	1 (1.2)	0.02
Noninvasive mechanical ventilation	**28 (7.4)**	23 (7.8)	5 (6.1)	0.81
Vasoactive infusions	**14 (3.7)**	11 (3.7)	3 (3.7)	1.00
Extracorporeal membrane oxygenation^††^	**1 (0.3)**	1 (0.3)	0 (—)	1.00
Hospital length of stay, days, median (IQR) (1)^§§^	**2 (1–3)**	2 (1–3)	2 (1–3)	0.89
Died before discharge (1)^††,¶¶^	**1/376 (0.3)**	1/294 (0.3)	0 (—)	1.00
**Infants with no underlying health conditions (% of all infants)**	**291 (77.2)**	230 (78.0)	61 (74.4)	—
Intensive care unit admission	**63 (21.6)**	47 (20.4)	16 (26.2)	0.38
Critical illness**	**37 (12.7)**	32 (13.9)	5 (8.2)	0.28
Invasive mechanical ventilation	**18 (6.2)**	18 (7.8)	0 (—)	0.02
Noninvasive mechanical ventilation	**19 (6.5)**	16 (7.0)	3 (4.9)	0.77
Vasoactive infusions	**12 (4.1)**	10 (4.3)	2 (3.3)	1.00
Extracorporeal membrane oxygenation^††^	**1 (0.3)**	1 (0.4)	0 (—)	1.00
Hospital length of stay, days, median (IQR) (1)^§§^	**2 (1–3)**	2 (1–3)	2 (1–3)	0.66
Died before discharge (1)^††,¶¶^	**1/290 (0.3)**	1/229 (0.4)	0 (—)	1.00

* Infants were excluded from analysis if they were born to mothers who had received their most recent dose before pregnancy, received only 1 dose of an mRNA vaccine, received their most recent vaccine dose within 14 days of delivery, received only 1 dose of a viral vector vaccine, or whose vaccination status could not be verified, or timing of vaccination was unknown.

^†^ Maternal vaccination status was based on the last date of a COVID-19 mRNA vaccine dose: unvaccinated was defined as mothers who had not received any vaccine dose before or during pregnancy, and vaccinated was defined as mothers who received their last dose of a COVID-19 mRNA vaccine between the first day of pregnancy and 14 days before delivery. Among those vaccinated during pregnancy, mothers could have received ≥1 dose during pregnancy. Mothers could receive 1 dose of Ad.26.CoV2.S (Janssen [Johnson & Johnson]) vaccine before or during pregnancy and 1 dose of an mRNA vaccine during pregnancy. Mothers who received only 1 dose of an mRNA vaccine were considered partially vaccinated and were excluded from the analysis. Mothers whose last vaccine dose occurred before pregnancy were excluded from the analysis.

^§^ Infants were enrolled from 26 pediatric hospitals in 20 states, in all four U.S. Census Bureau regions. *Northeast*: Boston Children’s Hospital (Massachusetts) and Cooperman Barnabas Medical Center (New Jersey); *Midwest*: Akron Children’s Hospital (Ohio), Children’s Hospital Medical Center (Ohio), Children’s Hospital of Michigan (Michigan), Children’s Mercy Kansas City (Missouri), C.S. Mott Children’s Hospital (Michigan), Lurie Children’s Hospital (Illinois), Mayo Clinic (Minnesota), Minnesota Masonic (Minnesota), Nationwide (Ohio), and Riley Hospital for Children (Indiana); *South*: Arkansas Children’s Hospital (Arkansas), Children’s of Alabama (Alabama), Children’s Healthcare of Atlanta, Emory (Georgia), Children’s Hospital of New Orleans (Louisiana), Medical University of South Carolina Children’s Health (South Carolina), Monroe Carell Jr. Children’s Hospital at Vanderbilt (Tennessee), Texas Children’s Hospital (Texas), University of Mississippi Medical Center (Mississippi), and University of North Carolina at Chapel Hill Children’s Hospital (North Carolina); *West*: Children’s Hospital Colorado (Colorado), Children’s Hospital Los Angeles (California), University of California, San Francisco Benioff Children’s Hospital (California), University of California San Diego-Rady Children’s Hospital (California), and Primary Children's Hospital (Utah).

^¶^ Testing for statistical significance was conducted using a Fisher’s exact test. Wilcoxon rank-sum tests were used to compare length of stay.

** Critical illness was defined as an illness that led to life support (noninvasive or invasive mechanical ventilation, extracorporeal membrane oxygenation, or vasoactive infusions) or death. Infants with an indication of any of these events were considered to have critical illness.

^††^ The infant receiving extracorporeal membrane oxygenation was not the same as the infant who died. The infant receiving extracorporeal membrane oxygenation was aged <3 months, and the infant who died was aged ≥3 months. The infant missing survival status at discharge was still hospitalized at the time of analysis.

^§§^ Length of stay was calculated among infants alive at discharge (376 among all infants and 290 among infants with no underlying health conditions). The infant missing length of stay was still hospitalized at the time of the analysis.

^¶¶^ One infant missing information about survival status at discharge was still hospitalized at the time of analysis. The denominators for the total and unvaccinated columns were reduced by 1 to account for this missing data. The infant who died was aged ≥3 months.

## Discussion

During March 2022–May 2023, maternal receipt of ≥1 COVID-19 vaccine dose during pregnancy was associated with a reduced risk for COVID-19–related hospitalization among infants aged <6 months. Protection was similar to previous estimates of maternal VE during the early period of Omicron variant predominance (*4*,*5*), but point estimates were higher when the analysis was limited to infants aged <3 months. This finding aligns with at least one other study, which demonstrated increased protection among infants during the first 90 days of life (*6*). In the current report, among 377 infants hospitalized with laboratory-confirmed COVID-19, 295 (78%) were born to women who had never received a COVID-19 vaccine dose. Currently, COVID-19 mRNA vaccines are approved in the United States for all persons aged ≥6 months, and these findings indicate that maternal vaccination during pregnancy could help prevent COVID-19–related hospitalization in infants too young to be vaccinated, particularly during the first 3 months of life.

Since the winter of 2022, COVID-19–associated hospitalization rates in infants aged <6 months have been higher than hospitalization rates in any age group except adults aged ≥65 years (*7*). COVID-19–associated hospitalizations and severe outcomes have occurred among predominantly healthy infants: among those aged <6 months hospitalized during March 20–August 31, 2022, 76% were previously healthy (*7*). Similarly, in the current report, previously healthy infants accounted for 77% of case-patients, with critical illness occurring in 13%. Maternal vaccination, including receipt of a third dose during pregnancy, has been associated with reduced risk for infant hospitalization (*4**–**6*). Further, maternal vaccination during pregnancy has not been associated with increased risk for adverse pregnancy and infant outcomes (*8*). Together, these data highlight the importance of early-life protection from severe COVID-19 outcomes through maternal vaccination.

### Limitations

The findings in this report are subject to at least six limitations. First, this investigation was not sufficiently powered to assess VE against hospitalizations attributed to specific Omicron subvariants. Second, the sample size was too small to assess VE with precision by vaccine manufacturer, dose number, receipt of bivalent doses, or timing of vaccination during pregnancy. Third, the analysis did not account for previous infection status among women before or during pregnancy, and infection-induced antibodies could provide some protection against infant COVID-19–related hospitalization. Fourth, the analysis did not collect information on maternal characteristics and protective behaviors, which are potential uncontrolled confounders. Fifth, maternal breastfeeding, which can confer maternal COVID-19 antibodies to the infant (*9*), could not be assessed because of the high proportion of missing interview responses. Finally, information on maternal vaccination status and infant race and ethnicity was collected via self-report for a few participants, potentially resulting in differential misclassification.

### Implications for Public Health Practice

Maternal receipt of ≥1 COVID-19 vaccine dose during pregnancy was associated with reduced odds of COVID-19–related hospitalization among infants aged <6 months, particularly among those aged <3 months. Additional evaluations should examine VE of maternal receipt of updated COVID-19 vaccines and the impact of potential waning immunity in infants aged ≥3 months. Expectant mothers should be counseled to remain current with COVID-19 vaccination to protect themselves and their infants from hospitalization and severe outcomes associated with COVID-19.

## Footnotes

* These authors contributed equally to this report.

† These senior authors contributed equally to this report.

§ Infants were enrolled from 26 pediatric hospitals in 20 states, in all four U.S. Census Bureau regions. *Northeast*: Boston Children’s Hospital (Massachusetts) and Cooperman Barnabas Medical Center (New Jersey); *Midwest*: Akron Children’s Hospital (Ohio), Children’s Hospital Medical Center (Ohio), Children’s Hospital of Michigan (Michigan), Children’s Mercy Kansas City (Missouri), C.S. Mott Children’s Hospital (Michigan), Lurie Children’s Hospital (Illinois), Mayo Clinic (Minnesota), Minnesota Masonic (Minnesota), Nationwide (Ohio), and Riley Hospital for Children (Indiana); *South*: Arkansas Children’s Hospital (Arkansas), Children’s of Alabama (Alabama), Children’s Healthcare of Atlanta, Emory (Georgia), Children’s Hospital of New Orleans (Louisiana), Medical University of South Carolina Children’s Health (South Carolina), Monroe Carell Jr. Children’s Hospital at Vanderbilt (Tennessee), Texas Children’s Hospital (Texas), University of Mississippi Medical Center (Mississippi), and University of North Carolina at Chapel Hill Children’s Hospital (North Carolina); *West*: Children’s Hospital Colorado (Colorado), Children’s Hospital Los Angeles (California), University of California, San Francisco Benioff Children’s Hospital (California), University of California San Diego-Rady Children’s Hospital (California), and Primary Children’s Hospital (Utah).

¶ Infants were hospitalized outside of their birth hospitalization.

** Infant receipt of breast milk was missing for 45% of respondents and was not included in the model; infant testing for coinfections was missing for 60% of infants and was not included in the model.

†† 45 C.F.R. part 46.102(l)(2), 21 C.F.R. part 56; 42 U.S.C. Sect. 241(d); 5 U.S.C. Sect. 552a; 44 U.S.C. Sect. 3501 et seq.

¶¶ One infant death before hospital discharge occurred in an infant aged ≥3 months whose mother was unvaccinated during pregnancy.
